# The enhancement and suppression of immersion mode heterogeneous ice-nucleation by solutes

**DOI:** 10.1039/c7sc05421a

**Published:** 2018-03-27

**Authors:** Thomas F. Whale, Mark A. Holden, Theodore W. Wilson, Daniel O'Sullivan, Benjamin J. Murray

**Affiliations:** a School of Earth and Environment , University of Leeds , Leeds , LS2 9JT , UK . Email: t.f.whale@leeds.ac.uk; b School of Chemistry , University of Leeds , Leeds , LS2 9JT , UK; c School of Physics and Astronomy , University of Leeds , Leeds , LS29JT , UK

## Abstract

Heterogeneous nucleation of ice from aqueous solutions is an important yet poorly understood process in multiple fields, not least the atmospheric sciences where it impacts the formation and properties of clouds. In the atmosphere ice-nucleating particles are usually, if not always, mixed with soluble material. However, the impact of this soluble material on ice nucleation is poorly understood. In the atmospheric community the current paradigm for freezing under mixed phase cloud conditions is that dilute solutions will not influence heterogeneous freezing. By testing combinations of nucleators and solute molecules we have demonstrated that 0.015 M solutions (predicted melting point depression <0.1 °C) of several ammonium salts can cause suspended particles of feldspars and quartz to nucleate ice up to around 3 °C warmer than they do in pure water. In contrast, dilute solutions of certain alkali metal halides can dramatically depress freezing points for the same nucleators. At 0.015 M, solutes can enhance or deactivate the ice-nucleating ability of a microcline feldspar across a range of more than 10 °C, which corresponds to a change in active site density of more than a factor of 10^5^. This concentration was chosen for a survey across multiple solutes–nucleant combinations since it had a minimal colligative impact on freezing and is relevant for activating cloud droplets. Other nucleators, for instance a silica gel, are unaffected by these ‘solute effects’, to within experimental uncertainty. This split in response to the presence of solutes indicates that different mechanisms of ice nucleation occur on the different nucleators or that surface modification of relevance to ice nucleation proceeds in different ways for different nucleators. These solute effects on immersion mode ice nucleation may be of importance in the atmosphere as sea salt and ammonium sulphate are common cloud condensation nuclei (CCN) for cloud droplets and are internally mixed with ice-nucleating particles in mixed-phase clouds. In addition, we propose a pathway dependence where activation of CCN at low temperatures might lead to enhanced ice formation relative to pathways where CCN activation occurs at higher temperatures prior to cooling to nucleation temperature.

## Abstract 2

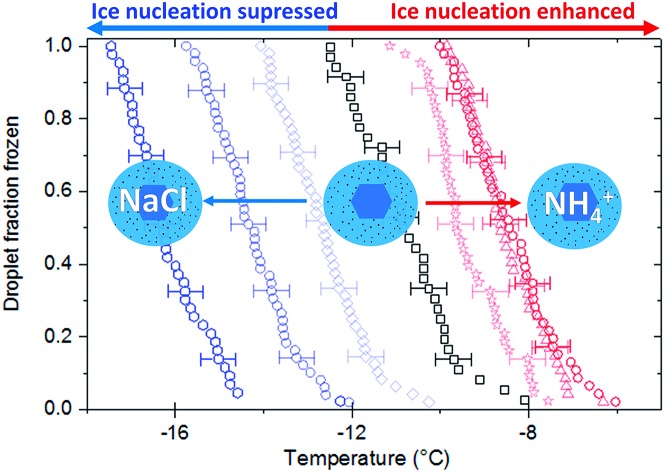
Heterogeneous nucleation of ice from supercooled liquid water by some atmospherically relevant nucleators is enhanced by ammonium salts and suppressed by alkali halides.

## Introduction

Ice nucleation is an important process in several fields. It has relevance to the atmosphere,^1^–^3^ cryopreservation of biological samples,^4^ freeze drying^5^ and freezing of foodstuffs.^6^ Ice nucleation in the natural environment will usually take place in aqueous solutions rather than pure water; it is known that this is the case in the atmosphere where, for example, cloud droplets in mixed-phase clouds are always composed of dilute solutions of a range of solutes. Each supermicron cloud droplet typically forms on a much smaller particle containing soluble hygroscopic material. A small proportion of these cloud droplets also contain ice-nucleating particles (INPs) and may go on to freeze heterogeneously if they become sufficiently cold.^7^ In contrast, ice clouds which form in the upper troposphere can form through the freezing of more concentrated submicron solution droplets called haze particles.^7^ Differences in the concentration and activity of INPs can have a substantial impact on weather and climate.^3^,^7^,^8^ Similarly, aqueous solutions used for cryopreservation, a process for which efficient ice nucleation is key,^4^ will usually contain a mixture of solutes to prevent damage to cells.^9^,^10^ However, most laboratory experiments on immersion mode^11^ ice nucleation are conducted with particles suspended in high-purity water with no additional solutes added.^1^–^3^

As such, the role of solute molecules in the freezing of liquid water is a process of fundamental and applied interest. In the case of homogenous ice nucleation (ice nucleation in the absence of any INPs), it is generally, accepted that the ‘water activity criterion’^12^ describes the change in nucleation temperature observed with the addition of solute molecules. That is to say, the shift in nucleation temperature observed with the addition of solute molecules can be calculated solely from the difference between the water activity of the solution in equilibrium with ice (the water activity at the melting point) and the water activity of the solution at the freezing temperature. This value was named Δ*a*_w_.^12^ This result was surprising when it was published as it might be expected the interfacial tension between solution and the critical nuclei, and the diffusion activation energy for a water molecule to cross the solution–ice interface are influenced by the nature of the solute molecules. Nevertheless, Koop *et al.*^12^ demonstrated that homogeneous freezing in the presence of 18 different aqueous solutes, ranging from inorganic salts like NaCl to organic solutes like glucose, is determined by water activity.

Much of the ice nucleation which occurs in the troposphere^3^ and all ice nucleation in cryopreservation systems^4^ is heterogeneous; induced by a substances other than water itself. The current paradigm for the impact of solutes on immersion mode heterogeneous ice nucleation was established by Zobrist *et al.*^13^ They conducted droplet freezing experiments using nonadecanol monolayers and dispersions of nanometer sized silica spheres, Arizona Test Dust (ATD) and AgI as ice nucleators. These nucleators were dispersed in various concentrations of a wide range of solute molecules which included salts such as LiCl, NaCl and (NH_4_)_2_SO_4_, organic compounds such as ethylene glycol and glycerol, organic acids and the polymer polyethylene glycol 300, among others. They found that an adapted form of the water activity criterion^13^ gave a satisfactory description of the freezing temperatures in experiments conducted in pure water and experiments conducted in the presence of solutes. Essentially, the freezing behavior of each nucleator could be described by a constant offset in water activity from the melting temperature with the size of the offset dictated by the nucleator. A characteristic shift in water activity could then be calculated for each of the four nucleators. For comparison, Δ*a*_w_ = 0.305 for homogenous nucleation whereas Δ*a*_w,het_ for heterogeneous nucleation ranged from 0.100 for the nonadecanol monolayers used to 0.195 for ATD. Several other studies with non-reactive solutes are consistent with the conclusion that heterogeneous freezing can be described by Δ*a*_w_.^14^–^18^

In contrast, there is evidence of acids causing reductions in the ice nucleation activity of certain mineral dusts that is greater than would be expected from the water activity of the acid solutions. These reductions are attributed to the destruction of, or coating of, ice-nucleating sites by the acid.^19^–^21^ Wilson and Haymet^22^ observed that heterogeneous nucleation caused by the glass wall of the apparatus used in their study was shifted to colder temperatures in solutions of 0–1 M glucose and NaCl by roughly twice the magnitude of the shift in melting temperature for these solutions. This is more of a shift than would be anticipated from the change in water activity of the solutions.

Reischel and Vali^23^ performed a study of the effects of 0.01 M, 0.1 M and 1 M solutions of 22 different salts on four different nucleators. The nucleators were kaolin, leaf-derived nuclei (LDN), AgI and CuS. LDN (now thought to be the ice nucleation active bacteria *Pseudomonas Syringae*^24^) was not significantly impacted by any solutes. For all other nucleators the responses were complicated with enhancements and suppressions of ice nucleation much larger than would be expected from changes in water activity. Remarkably, they observed that for CuS, AgI and kaolin the presence of ammonium salts usually led to higher nucleation temperatures. Other nucleator/solute combinations, particularly the combination of LiI and kaolin, were also observed to lead to large enhancements in ice nucleation efficiency. Gobinathan and Ramasamy^25^ demonstrated that dissolved NH_4_I can enhance the ice nucleation activity of PbI_2_. Zobrist *et al.*^13^ also observed that in the presence of (NH_4_)_2_SO_4_, AgI exhibited enhanced nucleation properties. As their experimental technique involved synthesis of AgI in the presence of solute molecules they attributed this to changes in crystal habit caused by changes in concentration of Ag^+^ in the presence of (NH_4_)_2_SO_4_. Overall, there are enough exceptions to the water activity criterion approach for heterogeneous freezing in the literature to warrant further investigation.

## Materials and methods

We have tested the ice nucleation activities of various combinations of nucleators and solutes using the microlitre Nucleation by Immersed Particles Instrument (μl-NIPI). This instrument has been described in detail previously^26^ and used for a number of studies of immersion mode heterogeneous ice nucleation with nucleants relevant for this study.^26^–^29^ Briefly, 40 to 50 droplets with a volume of 1 μl are placed on a silanised glass coverslip and cooled using a Grant-Asymptote EF600 Stirling cryocooler. Freezing temperatures for each droplets are recorded using a digital camera.

In order to facilitate comparison of data presented here with other studies we have calculated the ice nucleation active surface-site density, *n*_s_(*T*), for most samples. Specific surface areas cannot be reasonably determined for humic acid, hence *n*_s_(*T*) cannot be determined. For the other materials, *n*_s_(*T*) can be calculated using:^11^1
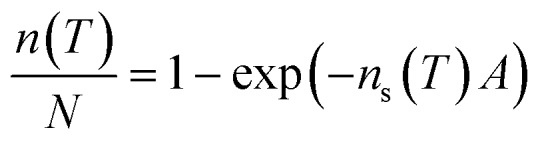
where *n*(*T*) is the number of droplets frozen at temperature (*T*), *N* is the total number of droplets in the experiment and *A* is the surface area of nucleant per droplet. *n*_s_(*T*) is a site specific measure of ice nucleation efficiency which does not account for the effects of cooling rate (*i.e.* time) dependence.^30^ All experiments here were conducted with the same cooling rate (1 °C min^–1^). Uncertainty in *n*_s_(*T*) was calculated using simulations of possible site distributions propagated with the uncertainty in surface area of nucleator per droplet as described in Harrison *et al.*^31^ Temperature uncertainty for μl-NIPI has been estimated to be ±0.4 °C.^26^

We also conducted ice nucleation experiments by placing individual chips of a feldspar rich rock, which were approximately 1 mm along the longest dimension, onto a hydrophobic slide. We then pipetted 1 μl MilliQ water droplets onto them and conducted freezing experiments using μl-NIPI as usual. We removed the rock chips from the water droplets, dried them and repeated the experiment in a 0.015 M solution of (NH_4_)_2_SO_4_.

For this study the solutes we used were KCl, NaCl, NaI, (NH_4_)Cl, (NH_4_)_2_SO_4_ and (NH_4_)OH. These compounds were purchased from Sigma-Aldrich. Previous studies of the impact of solutes on heterogeneous freezing have focused on relatively concentrated solutions droplets relevant for cirrus conditions where there is a significant colligative melting point depression.^13^–^15^,^17^ In contrast, we have investigated sufficiently dilute solutions that the colligative depression of melting point from that of pure water is smaller than, or comparable to, the temperature uncertainty of μl-NIPI. For the 0.015 M solutions the melting point depression is always less than 0.1 °C (0.04 °C, 0.04 °C and 0.06 °C, for NaCl, NH_4_Cl and KCl^32^ and 0.6 °C for (NH_4_)_2_SO_4_ 33 and for the 0.15 M solutions it is comparable to our temperature uncertainty (0.5 °C for NaCl^32^ and 0.6 °C for (NH_4_)_2_SO_4_.^33^ The water activity of these solutions is therefore approximately unity at the melting temperature and since the temperature dependence of water activity of inorganic solutions is typically very weak,^33^,^34^ it is expected that the activity at freezing will also be approximately unity. Hence, colligative effects on the freezing point are expected to be, at most, comparable to the temperature uncertainty of the μl-NIPI.

A range of nucleators have been used for this study. BCS376 microcline, which was initially characterized by Atkinson *et al.*^28^ was obtained from the Bureau of Analysed Samples. It contains 76.6% alkali feldspar, 16.7% plagioclase feldspar and 3.9% quartz. Eifel sanidine was first tested by Whale *et al.*^35^ and is pure alkali feldspar which lacks perthitic texture and therefore is an inefficient nucleator. Humic acid (leonardite) was purchased from the International Humic Substances Society. Its ice-nucleating activity has been tested previously.^36^ Quartz was purchased from Sigma Aldrich. The sample used was found to be 98.4% alpha-quartz by Atkinson *et al.*^28^ Nanoporous amorphous silica gel was purchased from Sigma-Aldrich (214396) and ultra-fine Arizona Test Dust (ATD) was purchased from Powder Technology inc. Solutions. Suspensions of these nucleators were made up gravimetrically.

**Fig. 1:**
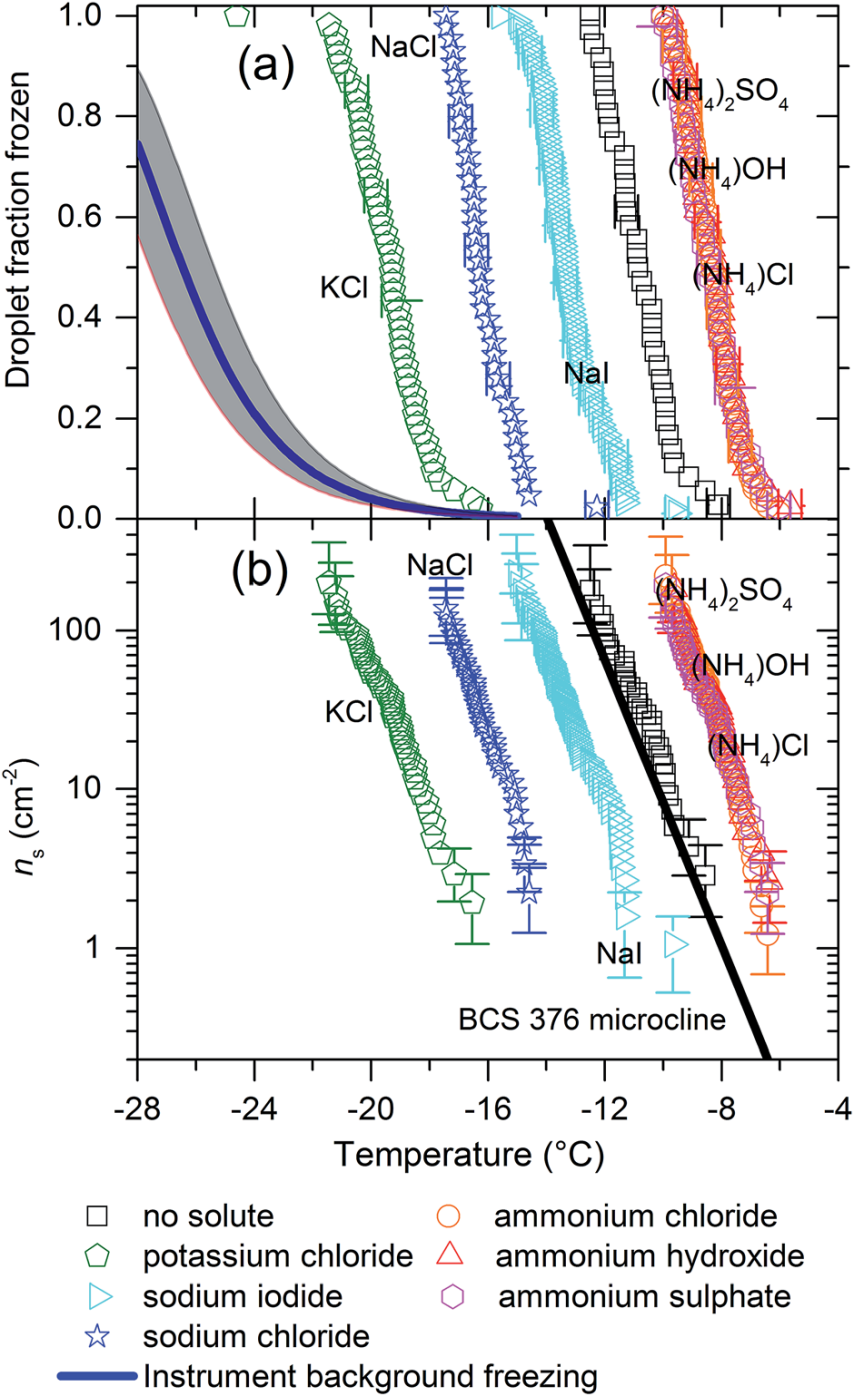
(a) Fraction frozen curves and (b) *n*_s_(*T*) values (bottom) for 0.1 wt% of BCS 376 microcline suspended in 0.015 M solutions of various solutes. The black line is the paramaterisation for the ice nucleation activity of BCS376 microcline from Atkinson *et al.*^28^ The concentration of solute used produces a freezing point depression of less than 0.1 °C. The water activity of these solutions is very close to 1 so no significant depression in nucleation temperature would be expected given our experimental temperature uncertainty is ±0.4 °C. All three ammonium compounds cause ice to nucleate 3 °C warmer while the three alkali halides produced freezing point depressions ranging from 2.5 °C for NaI to 8.5 °C for KCl. A fit for the background freezing of the instrument using pure MilliQ water from Umo *et al.*^38^ The shaded region shows 95% confidence intervals for the fit.

The instrument we have used for this study, μl-NIPI, is not capable of investigating homogeneous freezing, at least in the configuration used here. Background heterogeneous ice nucleation, either from the hydrophilic slides or impurities in the MilliQ water source cause ice nucleation to occur starting at warmer temperatures than those predicted by classical nucleation theory (CNT).^37^ A background freezing curve for MilliQ water μl-NIPI containing no added particles or solutes, is shown in Fig. 1.^38^ Experiments conducted with 0.015 M solutions of KCl and NH_4_Cl did not change the background freezing curve observed. Hence, we conclude that the background freezing did not interfere with observed nucleation by the added nucleants discussed in the next section.

## Results

In Fig. 1 we present the results of experiments where we froze 0.1 wt% suspensions of BCS 376 microcline in 0.015 M solutions of potassium chloride, sodium iodide, sodium chloride, ammonium hydroxide, ammonium chloride, ammonium sulphate and pure water using μl-NIPI. In this plot, and all others, the nucleant suspended in water without additional salts is shown in black. All three ammonium salts enhanced ice nucleation, leading to freezing temperatures about 3 °C warmer than the pure water case. The extent of enhancement was identical in all cases. In contrast, the presence of alkali halides led to colder freezing temperatures. The deactivation in all cases was much greater than would be expected from the water activity criterion^13^ (which would be less than 0.1 °C as discussed in materials and methods above).

Clearly, ice nucleation by BCS 376 microcline is sensitive to the identity of solute ions and does not follow the water activity criterion at the solute concentration investigated here. The variation in freezing point depressions caused by the alkali metal halides has the order KCl > NaCl > NaI. The effect is striking, with KCl causing a decrease in freezing temperatures of about 8 °C and ammonium salts enhancing freezing by 3 °C, a spread of more than 10 °C. Taking dlog(*n*_s_)/d*T* from the BCS376 ice nucleation activity parameterization in Atkinson *et al.*,^28^ a shift of 10 °C corresponds to a change in *n*_s_ of a factor of about 10^5^.

**Fig. 2:**
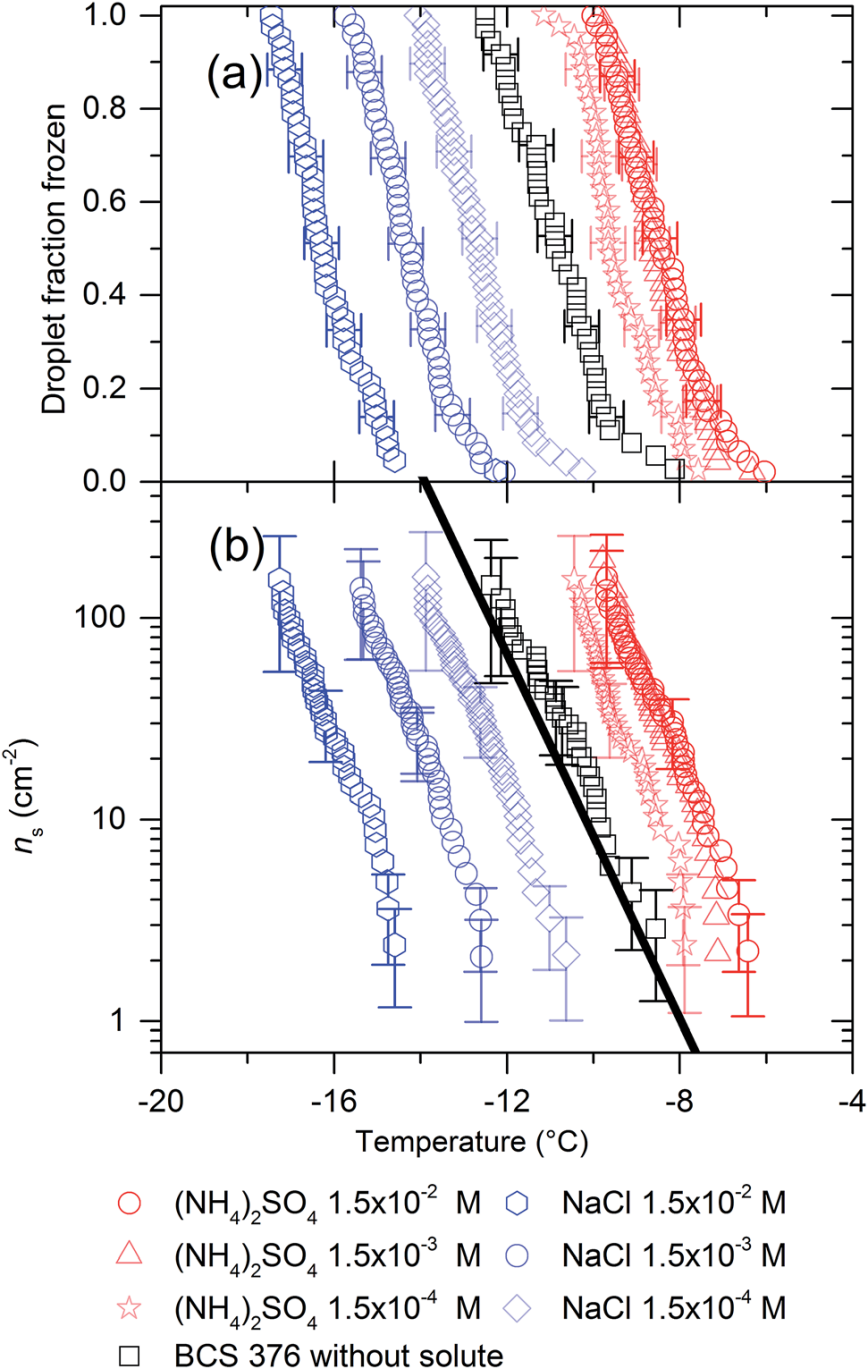
(a) Fraction frozen curves and (b) *n*_s_(*T*) values for 0.1 wt% of BCS 376 microcline suspended in various concentrations of (NH_4_)_2_SO_4_ and NaCl. The black line is the paramaterisation for the ice nucleation activity of BCS376 microcline from Atkinson *et al.*^28^ It can be seen that reduced concentrations of solutes lead to smaller impacts on freezing temperatures.

We have also examined the effect of varying concentration of (NH_4_)_2_SO_4_ and NaCl on nucleation by BCS 376 microcline, the results are presented in Fig. 2. For these experiments we reduced concentrations of the solutes by factors of 10 and 100. For NaCl the magnitude of the reduction in freezing temperature decreased with reducing salt concentration. For (NH_4_)_2_SO_4_ there was little difference in the level of enhancement between the 1.5 × 10^–2^ M and 1.5 × 10^–3^ M solution while the enhancement in freezing activity was smaller for the 1.5 × 10^–4^ M solution, around 1.5 °C rather than 3 °C. This strongly suggests there is a limit to the extent to which the presence of ammonium salts can enhance ice nucleation efficiency, particularly when viewed in combination with the data in Fig. 1 showing that all ammonium salts lead to an equal enhancement of ice nucleation.

**Fig. 3:**
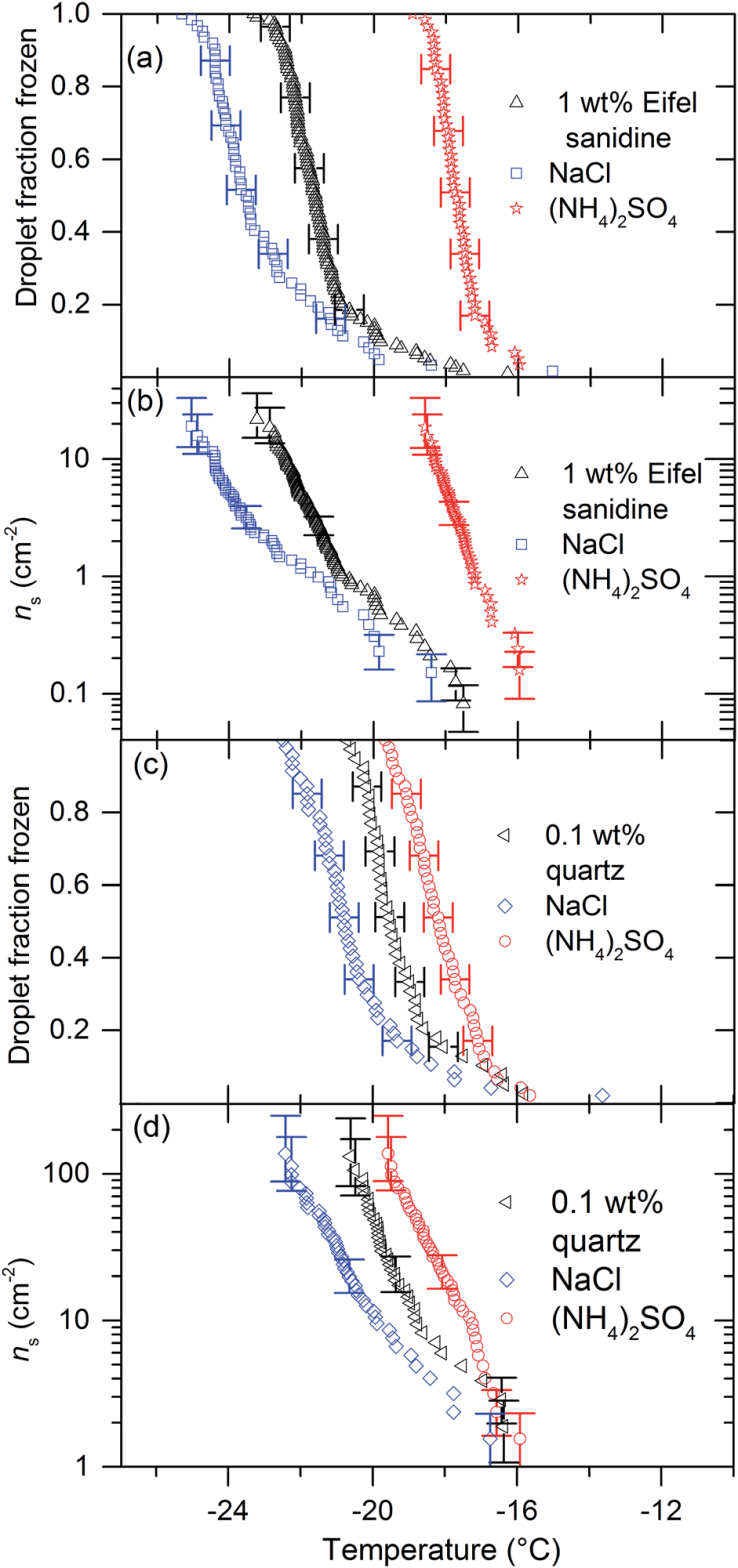
The impact of 0.015 M NaCl and (NH_4_)_2_SO_4_ on ice nucleation by quartz and Eifel sanidine. (a) Droplet fraction frozen against temperature for a 1 wt% Eifel sanidine suspension with data showing the impact on freezing temperature of 0.015 M NaCl and 0.015 M (NH_4_)_2_SO_4_. (b) *n*_s_(*T*) values for the fraction frozen data in panel (a). (c) Droplet fraction frozen against temperature for a 0.1 wt% quartz suspension with data showing the impact on freezing temperature of 0.015 M NaCl and 0.015 M (NH_4_)_2_SO_4_. (d) *n*_s_(*T*) values for the fraction frozen data in panel (c). For these nucleators the presence of NaCl reduces nucleation temperatures while the presence of (NH_4_)_2_SO_4_ increases nucleation temperatures.

**Fig. 4:**
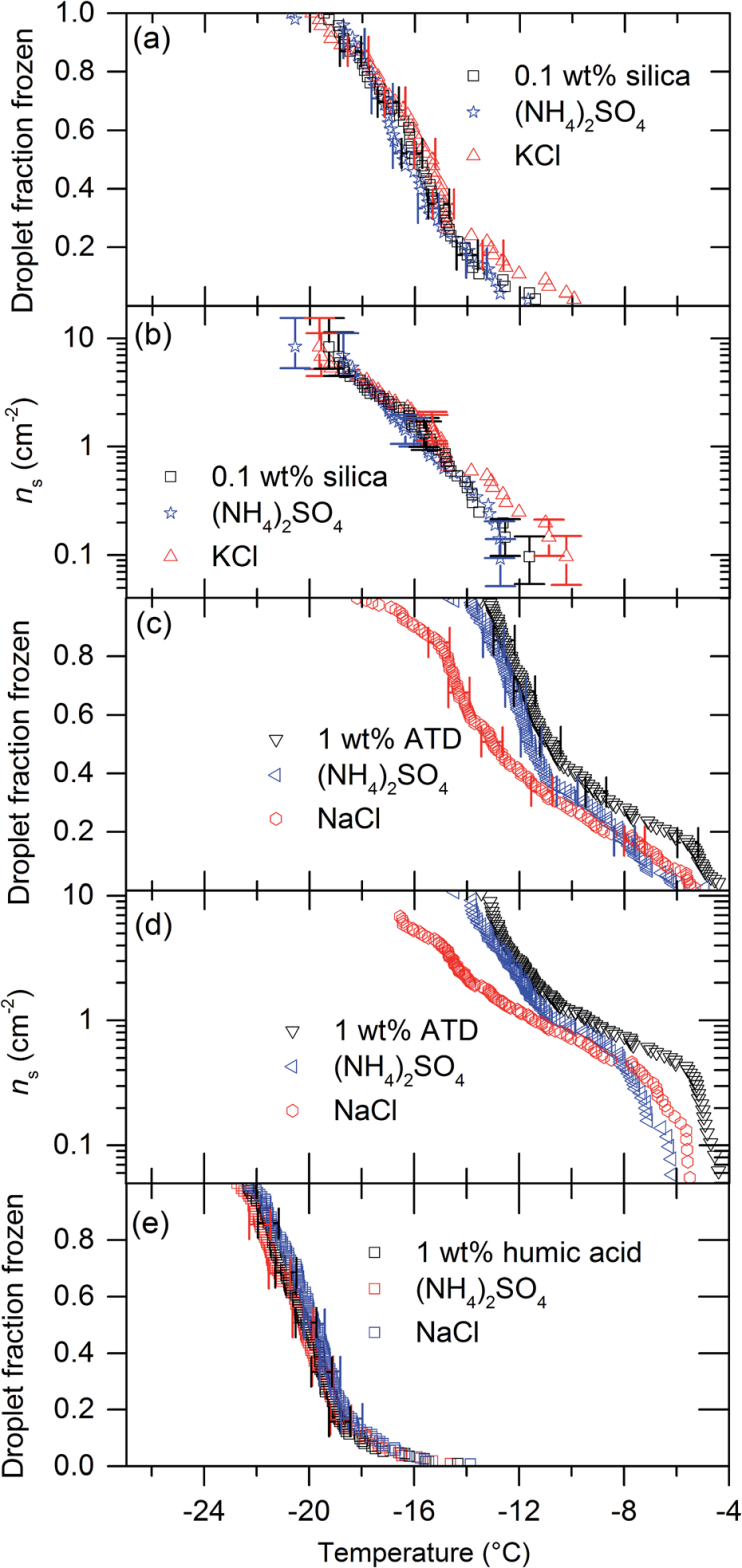
The impact of 0.015 M KCl and (NH_4_)_2_SO_4_ on ice nucleation by silica, humic acid and ATD. (a) Droplet fraction frozen against temperature for a 0.1 wt% silica suspension with data showing the impact on freezing temperature of 0.015 M NaCl and 0.015 M (NH_4_)_2_SO_4_. (b) *n*_s_(*T*) values for the fraction frozen data in panel (a). (c) Droplet fraction frozen against temperature for a 0.1 wt% ATD suspension with data showing the impact on freezing temperature of 0.015 M KCl. (d) *n*_s_(*T*) values for the fraction frozen data in panel (c). (e) Droplet fraction frozen against temperature for a 1 wt% humic acid suspension with data showing the impact on freezing temperature of 0.015 M KCl.

We tested five other nucleators with 0.015 M solutions of (NH_4_)_2_SO_4_, KCl and NaCl. Fig. 3 and 4 show the results of these experiments. Eifel sanidine and quartz qualitatively showed the same response as BCS376, *i.e.* (NH_4_)_2_SO_4_ increased ice nucleation temperatures while NaCl and KCl reduced them (Fig. 3) whereas humic acid and silica gel were unaffected by the presence of solutes (Fig. 4). ATD showed a mixed response to the presence of solutes. While addition of (NH_4_)_2_SO_4_ did not significantly change freezing temperatures of ATD droplets, addition on KCl did reduce freezing temperatures at colder temperatures. We observed no temperature shift for experiments conducted using silica gel and humic acid as nucleators. The observation that solutes can influence nucleation by some materials, but not others is particularly interesting and will be addressed later. Also, the fact that the freezing temperatures do not shift for some materials confirms that the water activity of the solutions used in those cases is indeed equal to unity to within the sensitivity of μL-NIPI.

The impact of solutes on ice nucleation by silica and humic acid have been tested previously so some comparison to literature data is possible. The silica gel we have tested is amorphous, as were the silica balls tested by Zobrist *et al.*^13^ These two materials may therefore nucleate ice *via* a similar mechanism and our results are indeed compatible with the results of Zobrist *et al.*^13^ and the water activity criterion.

The humic acid we have tested is probably similar to humic substances tested by Knopf and Alpert^14^ and Rigg *et al.*^15^ These studies showed that the water activity criterion described ice nucleation at lower water activities and our results are in agreement – we saw no difference in freezing temperatures for these nucleators. These previous studies did not look at quartz or feldspars where we have observed a strong dependence on solute.

Droplets containing ATD were frozen with various solutes by Zobrist *et al.*^13^ Their results are compatible with the water activity criterion, which fits with the results we have produced for (NH_4_)_2_SO_4_ but not with the results for KCl. It is interesting that only the lower temperature points for the ATD/KCl experiments are impacted strongly. It is not known what component of ATD is responsible for its ice-nucleating ability. It is certainly a complex mixture of many minerals.^39^,^40^ It is possible that the much larger droplets we used for this study compared to those used in Zobrist *et al.*^13^ mean that our experiments have sampled rarer active sites than Zobrist *et al.*^13^ which might have a different response to the presence of solutes.

**Fig. 5:**
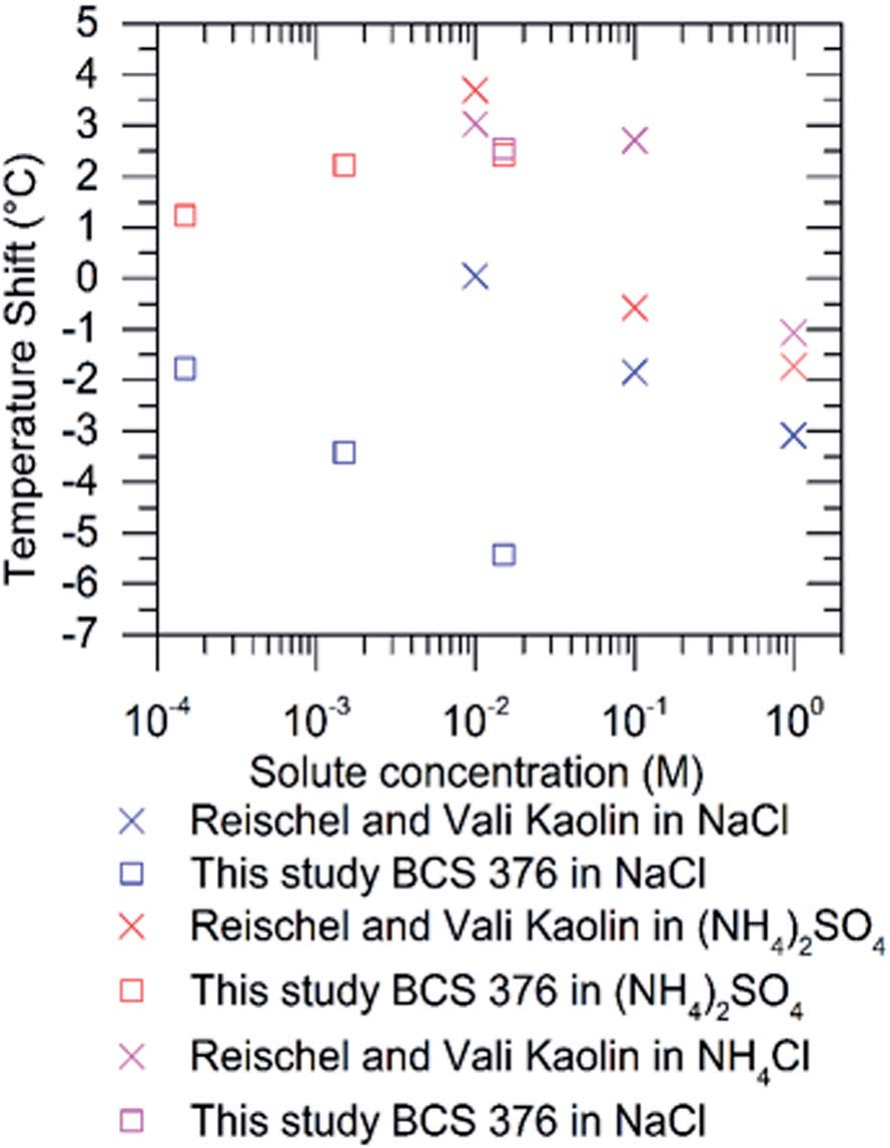
Comparison of shifts in true supercooling induced by (NH_4_)_2_SO_4_, NH_4_Cl and NaCl in droplets containing kaolin from Reischel and Vali^23^ with shifts for BCS 376 feldspar from this study. Shifts for the present study were calculated from the difference in the temperature at which 50% of droplets froze between the pure water experiment and the experiment with the solute. 0.01 M solutions of the ammonium salts lead to similar enhancements in both studies. We obtained a different temperature shift for 0.01 M NaCl, although it should be noted that the nucleators are different.

Reischel and Vali^23^ observed enhancement of ice nucleation by ‘kaolin’ in the presence of ammonium salts. It is possible that this material contained some feldspar as clays such as kaolinite are very often produced from weathering of feldspars.^41^ Indeed, kaolinite samples have been shown to contain significant amounts of feldspar.^28^ Hence, we have compared the impacts of (NH_4_)_2_SO_4_, NH_4_Cl and NaCl on kaolin from Reischel and Vali^23^ and BCS 376 from this study in Fig. 5. Interestingly, similar shifts to warmer freezing temperatures (about 2.5 °C to 3.5 °C) were observed in both studies for concentrations of (NH_4_)_2_SO_4_ and NH_4_Cl of approximately 0.01 M. The other direct point of comparison, 0.01 M NaCl, does not agree well. We observed a shift of 5.5 °C to colder temperatures while Reischel and Vali^23^ observed very little shift. It is difficult to compare to any other literature data as there is no commonality in the nucleators used.

Nevertheless, it is clear that at the concentrations we have investigated ammonium salts can enhance ice nucleation and alkali halides can inhibit it. Our work suggests that some nucleators are not affected by solutes however it is clear from Reischel and Vali^23^ that varying concentrations of solutes can have unpredictable impacts on nucleation temperatures so it is possible that higher or lower concentrations of solutes would alter freezing temperatures unpredictably. Equally, as most data supporting the water activity criterion comes from more concentrated solutions it is conceivable that at higher concentrations the effects we have observed will become less significant and that colligative effects (probably described by the water activity criterion) start to dominate changes in freezing temperatures due to solutes.

It has been suggested that aggregation of particles in droplet freezing experiments, leading to a loss of particles from suspension and a reduction of surface area within aggregates of particles, may be responsible for discrepancies between different instruments used for measuring ice nucleation.^42^ It is well known that aqueous salts can change the rate at which particles aggregate and the size of aggregates, hence it is conceivable that aggregation could be enhanced or inhibited on adding salts leading to changes in ice-nucleating activity.

**Fig. 6:**
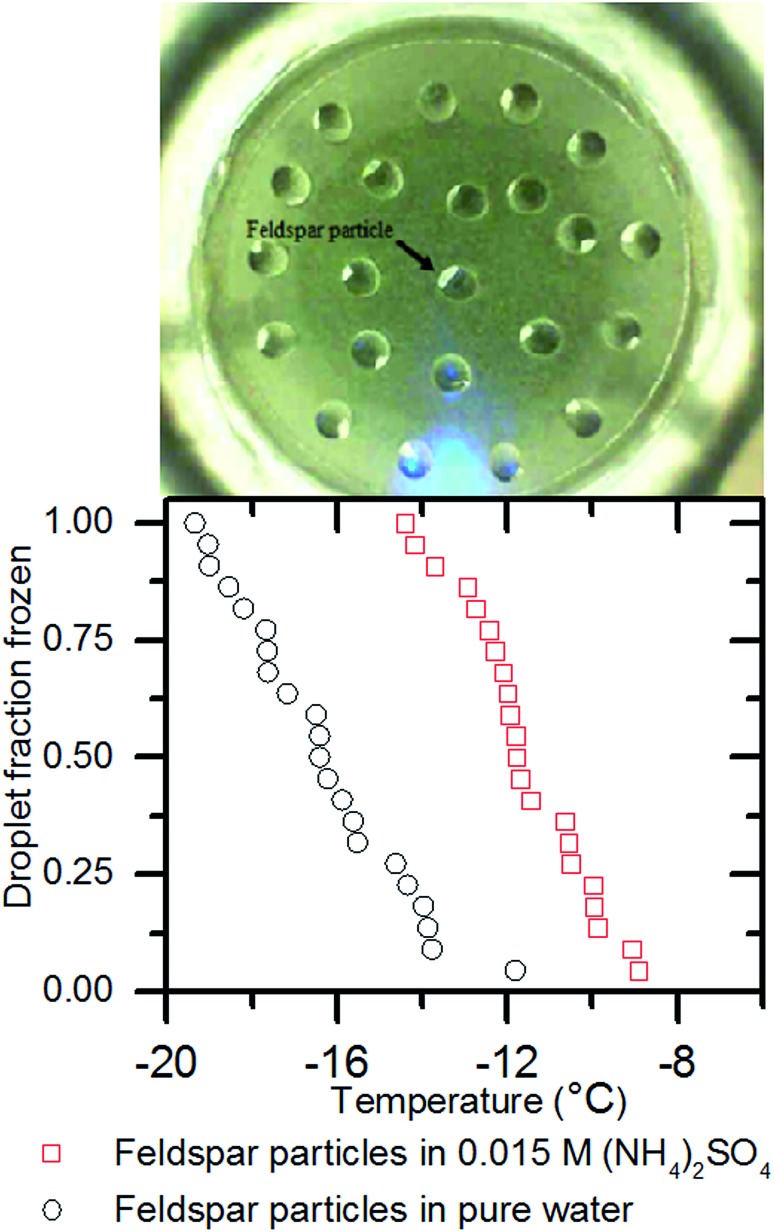
The top panel is a photograph of chips of feldspar immersed in water droplets in the μl-NIPI system. The white parts of the droplets are the immersed chips. When these water droplets were replaced by a 0.015 M ammonium sulphate solution ice nucleation was enhanced. The bottom panel shows the fraction of droplets frozen against temperature with and without ammonium sulphate.

To test this we took individual chips of a feldspar rich rock of approximately 1 mm diameter and placed them onto a hydrophobic slide. We then pipetted 1 μl Milli-Q water droplets onto them and conducted a freezing experiment using μl-NIPI as usual. The left hand panel of Fig. 6 shows an image of the experiment. In the left hand panel of Fig. 6 it can be seen that the median freezing temperature was –16.4 ± 0.4 °C. We removed the rock chips from the water droplets, dried them and repeated the experiment in a 0.015 M solution of (NH_4_)_2_SO_4_. Median freezing temperature shifted to –11.8 ± 0.4 °C. This demonstrates that the ice nucleation enhancing effect of (NH_4_)_2_SO_4_ does not depend on the nucleator being in powder form and that the solute effect on ice nucleation is not related to particle aggregation.

As there is no way in which aggregation can occur in this system we conclude that aggregation of particles is not the reason for solute effects on ice nucleation we have observed. We are therefore left to consider other mechanisms by which solutes may impact ice nucleation in ways beyond the water activity criterion.

## Discussion

These results have significant and obvious atmospheric implications. (NH_4_)_2_SO_4_ and NaCl are common components of atmospheric aerosol,^43^ and this work shows that even dilute solutions of these salts have the potential to substantially impact ice nucleation rates in cloud droplets which are nucleated by feldspar, which is thought to play a significant role in ice nucleation in mixed-phase clouds.^28^,^44^,^45^ For instance, a 0.5 μm ammonium sulphate particle which is activated and grows to a diameter of 10 μm, a typical cloud droplet size, would have an ammonium sulphate concentration of approximately 0.002 M, which is within the concentration range we have investigated here. The mixing state of aerosol will clearly play a key role in any interaction between dissolved ammonium sulphate in cloud droplets and feldspar (or other mineral dusts) aerosol suspended in those same droplets. If the two species do not end up in the same droplet there can be no interaction. However, there is already evidence that elevated ammonium sulphate concentrations correlate with ice-nucleating particle (INP) concentrations in plumes of Saharan desert dust sampled from a mountain top-observatory in Europe.^46^ Conversely, the based on our results we would anticipate NaCl internally mixed with K-feldspar to reduce the activity of feldspar in the atmosphere. This is a topic which should most certainly be pursued in the future.

**Fig. 7:**
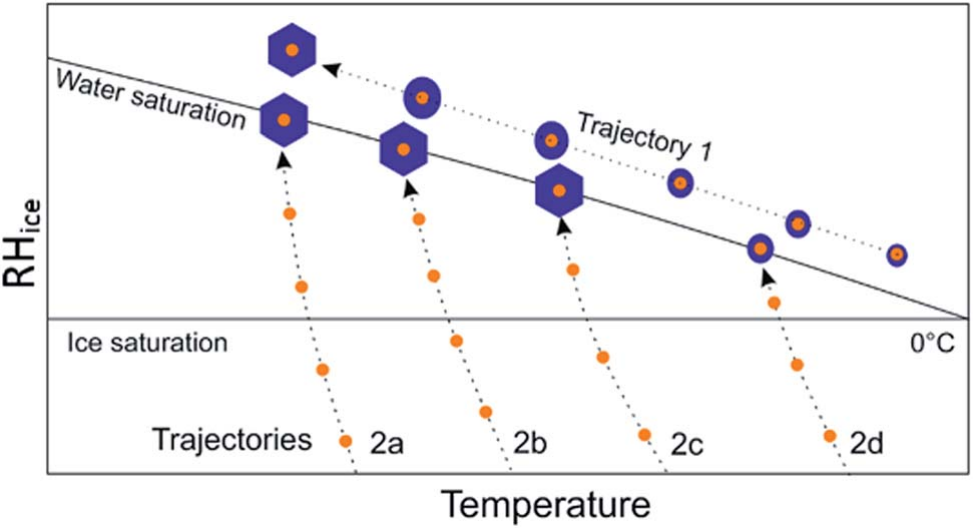
Schematic diagram showing different pathways to ice nucleation for mineral dust particles internally mixed with ammonium salts (small orange circles). Ice crystals are represented by blue hexagons and water droplets by blue circles. Trajectory 1 shows a pathway of the type observed in cold-stage droplet freezing style experiments or cloud droplets which form high temperatures and then cool. Trajectories 2a–d are typical of situations where particles approach water saturation from sub-saturated conditions, as would often be found in cloud chamber style experiments.

The solute effect we report here may result in a pathway dependence of nucleation where nucleation along one RH-temperature pathway may be different to nucleation along another pathway. We illustrate this in Fig. 7 for the case of a mineral dust particle internally mixed with an ammonium salt. Trajectory 1 illustrates a pathway where a water droplet condenses on an aerosol particle at high temperature and grows as temperature decreases. In this case, any ammonium salt present is likely to be very dilute by the time the water droplet gets cold enough to freeze and so will not influence freezing temperatures. This trajectory is analogous to that used by most cold-stage droplet freezing style experiments as well as clouds where droplet formation occurs at temperatures well above freezing temperatures. In trajectories 2a–c however, where the pathway approaches water saturation from the subsaturated regime, we suggest that dust particles will experience short periods exposed to elevated concentrations of ammonium salts as they take up water at around water saturation. This trajectory is relevant for instruments such as expansion chambers and also a subset of clouds. Given our experimental results we propose that this trajectory will result in ice nucleation at warmer temperatures and therefore an enhanced *n*_s_. If experiments are conducted with ammonium salts, or other soluble components which enhance nucleation, internally mixed with the ice nucleators under test this could lead to discrepancies between different instruments for measuring ice nucleation. Discrepancies have been observed between certain dry-dispersed aerosol instruments and cold-stage droplet freezing instruments, where the cold stage instruments tended to produce smaller *n*_s_ values at the same temperature.^47^ This is consistent with the pathway dependence proposed here; this warrants further investigation. Similarly, in clouds, the same INPs could potentially nucleate ice with different efficiencies depending on the pathway.

Equally, this work shows that consideration of solute content may be of vital importance for controlling ice nucleation in cryopreservation systems, which often determines the effectiveness of these procedures. In the presence of alkali halides attempts at nucleation of ice in cryopreservation vessels, which is known to be of critical importance for cell survival,^4^ may be compromised. NaCl is universally present in the media used for cell culture and cryopreservation so this is something which may be considered for future work on ice nucleation in cryobiology.

The underlying concern for all applied areas where knowledge about ice nucleation is of interest (notably the atmosphere and cryobiology as discussed above) is that there is very little understanding of why any particular substance should nucleate ice efficiently. Traditionally, it has been assumed that a mixture of strong hydrophilicity and good lattice match to ice will cause a substance to nucleate ice efficiently.^48^ More recent work has shed a great deal of doubt on this and certainly shows the story is far too complicated to be analysed in terms of these two parameters alone.^31^,^35^,^49^–^54^

The results we present here suggest an avenue to help unpick the tricky problem of the mechanisms by which heterogeneous nucleation of ice from liquid water occurs. Clearly, various solutes influence different nucleators differently with respect to their ice nucleation properties. As such it may be possible to classify nucleators by their responses to the presence of solutes.

It is known that ice nucleation by feldspar minerals is strongly influenced by their ‘microtexture’ and most probably by associated topographical features.^35^ In general, the site-specific nature of ice nucleation by most nucleators which have been tested for time dependent properties^30^,^55^,^56^ implies that there must be a strong spatial heterogeneity in the nucleating ability of their surfaces, which is most readily interpreted as variable surface topography. It is unlikely that the presence of these unreactive solutes will alter the physical topography of ice nucleators on the timescales of the experiments presented (as opposed to reactive acids, such as H_2_SO_4_ which will react with and cause the dissolution of some surface components). Hence, it seems that the presence of solutes either directly influences the formation of critical nuclei or alters the active sites on the surface in ways which promote ice nucleation. This alteration could change the strength of interaction of the nucleator with liquid water or could conceivably subtly alter the spatial location of surface groups on the nucleator, giving a better surface for ice nucleation.

It has been shown that in computational models different surfaces can nucleate ice *via* different mechanisms.^52^,^57^ For instance, Fitzner *et al.*^52^ identified three different ways in which different surfaces could nucleate ice. These are: a typical lattice match scenario where the surface creates an in-plane template for an ice face, a mechanism whereby ice like structure is induced by appropriate structuring of the first two overlayers of a relatively rough (on an atomic scale) nucleator surface and a mechanism where strong interaction between the nucleator surface and water induces structure several water layers above the surface. It is likely that different ice nucleators in experimental systems nucleate ice in different ways and if the differing responses to the presence of solute correspond to different mechanism this might be a way to classify ice nucleators by mechanism.

In the light of these ideas about the how chemical effects can influence ice nucleation, we can see three broad categories of mechanism by which ammonium salts and alkali metal halides might influence ice nucleation by certain nucleators:

(1) Nucleant modification: replacement of cations with NH_4_^+^ in the nucleant surface enhances ice nucleation by altering surface properties in a way conducive to efficient ice nucleation. Conversely, insertion of alkali metal cations into surfaces does the reverse, inhibiting ice nucleation. Nucleators that cannot readily incorporate cations into their structures are not affected in the same way.

(2) Adsorption on the nucleant: adsorption of NH_4_^+^ onto the nucleator surface changes the strength of the interaction of water with the surface in a way favorable to ice nucleation. Conversely, adsorption of alkali metal cations to the surface inhibits ice nucleation or else metal cations fail to adsorb to the surface.

(3) Aqueous phase interactions: the interaction of aqueous NH_4_^+^ with water alters the nature of the critical ice nucleus in a way that encourages ice nucleation. Conversely, aqueous alkali metal cations disrupt critical nucleus formation. Different nucleators nucleate ice *via* different mechanisms, which are impacted differently by solutes. There are various potential mechanisms by which disruption or promotion of critical ice nuclei can occur, which also potentially apply to homogeneous ice nucleation. These are discussed below.

Any aqueous phase interaction which impacts heterogeneous ice nucleation might reasonably be expected to also play some role in homogeneous nucleation. Hence, it is perhaps surprising the water activity criterion appears to account for solute induced shifts in homogeneous ice nucleation temperatures. It might be expected that different solute species would influence interfacial tension between solution and the critical nuclei, and the diffusion activation energy for a water molecule to cross the solution–ice interface. For the solute compounds we have used in this study the seminal paper by Koop *et al.*^12^ found that the water activity criterion applies for homogeneous ice nucleation in the presence of several of the salts we have investigated here, (NH_4_)_2_SO_4_, NaCl, KCl and NH_4_Cl. As far as we are aware no data is available for homogeneous nucleation in the presence of NH_4_OH or NaI. Overall, it appears that the water activity criterion is a good approximation for homogeneous nucleation and exceptions to this have been attributed to contamination of the solute compounds used.^58^ However, recent molecular dynamics simulations have suggested that this might not be the case, finding that the presence of NaCl increases the interfacial free energy of the forming ice cluster thereby reducing the rate of ice nucleation.^59^,^60^ Similar molecular dynamics studies combining solute interactions with heterogeneous nucleators may shed light on the results presented in this paper.

We now make some observations about the physical and chemical characteristics of several of the nucleators in order to suggest some possible explanations for our observations and link to the three proposed options for the mechanism by which solutes influence ice nucleation.

• It is also known that NH_4_^+^ can substitute for the cations in feldspars to form ammonium feldspar^61^,^62^ so it may well be that some amount of substitution occurs in dilute ammonium solutions, altering the strength of water binding to the surface and impacting ice nucleation. It has been shown computationally that varying interaction strength of surface with water can have complex effects on nucleation.^52^ This seems a reasonable hypothesis which should be tested.

• The split and more complex response to solutes observed for ATD (nucleation is suppressed by both NaCl and (NH_4_)_2_SO_4_ at warmer temperatures, relatively unaffected by (NH_4_)_2_SO_4_ at colder temperatures and suppressed by NaCl at colder temperatures) suggests that the two effects are not necessarily caused by the same mechanism with reversed influences from ammonium salts and alkali halides.

• The chemical composition of silica is identical to that of quartz so it is interesting that quartz is apparently influenced by solute effects while silica is not. It may be that the regular crystal structure of quartz is more susceptible to inclusion of solute ions than the amorphous structure of silica. Sum-frequency vibrational spectroscopy has been used to show that ammonium can promote ordering of water at the surface of sapphire crystal^63^ and silica.^64^ These studies show that this occurs due to adsorption of NH_4_^+^ to aluminol and silanol groups on the surfaces of the sapphire and silica, respectively. Such groups are likely to occur on the surfaces of most of the nucleators we have studied here so adsorption of this type modifying the strength of interaction with water provides a plausible explanation for the effects we have observed.

• It is interesting to note that NH_4_^+^ is isostructural with H_3_O^+^, and is readily able to take the place of the hydronium ion in the ice lattice. Indeed, numerous past experiments have confirmed the strong propensity of this ion to be readily incorporated into water ice.^65^,^66^ It is currently unclear whether this fact is related to its enhancement effect on ice nucleation, and it will require further mechanistic studies to confirm.

• Finally, we note that the solute effect is observed for both Eifel sanidine and BCS 376 microcline. It has been shown that BCS 376 microcline nucleates ice more efficiently than Eifel sanidine and that this is most probably due to topographical features associated with boundaries between K and Na rich regions, which Eifel sanidine lacks, despite having similar crystallographic structure and chemical composition to other alkali feldspars.^35^ This suggests that the sites on the two kinds of feldspar have a similar nature as regards ice nucleation, even though they nucleate ice at different temperatures.

## Conclusions

Overall, we have shown that low (≤0.15 M) concentrations of ammonium salts and alkali halides can have a profound influence on the ice-nucleating efficiency of some nucleators, but not others. The current paradigm is that temperature shifts in heterogeneous nucleation temperatures due to solutes in a freezing solution depends entirely on the water activity of the solution, and does not depend on the specific solute species.^13^ It is clearly the case that solutes can impact heterogeneous ice nucleation temperatures substantially even at concentrations too low to have a significant impact on water activity, suggesting that the water activity criterion does not apply to all mechanisms of heterogeneous ice nucleation.

This work is potentially of great importance for understanding ice nucleation in mixed phase clouds, which have a profound impact on climate,^8^ suggesting that current understanding and modelling may be flawed in a significant way. Indeed, it has recently been shown that INP concentrations correlate with the concentration of ammonium sulphate in a region rich in mineral dusts near the Sahara desert suggesting that the presence of ammonium may be enhancing atmospheric ice nucleation in the natural environment.^46^

Regardless of applications, the solute effects on ice nucleation provides a potential route to differentiating mechanisms of immersion mode ice nucleation by different nucleators and therefore a route to understanding these mechanisms. Further work looking at different solutes, different nucleators and concentration dependences may help to unpick the puzzle.

## Conflicts of interest

There are no conflicts to declare.
