# Correction: The enhancement and suppression of immersion mode heterogeneous ice-nucleation by solutes

**DOI:** 10.1039/c8sc90139b

**Published:** 2018-07-12

**Authors:** Thomas F. Whale, Mark A. Holden, Theodore W. Wilson, Daniel O’Sullivan, Benjamin J. Murray

**Affiliations:** a School of Earth and Environment , University of Leeds , Leeds , LS2 9JT , UK . Email: t.f.whale@leeds.ac.uk; b School of Chemistry , University of Leeds , Leeds , LS2 9JT , UK; c School of Physics and Astronomy , University of Leeds , Leeds , LS2 9JT , UK

## Abstract

Correction for ‘The enhancement and suppression of immersion mode heterogeneous ice-nucleation by solutes’ by Thomas F. Whale *et al.*, *Chem. Sci.*, 2018, **9**, 4142–4151.



## 

The authors regret that in the original article they didn't acknowledge the main grant which supported this research. The missing grant was from the European Research Council (ERC, 713664 CryoProtect).

The Royal Society of Chemistry apologises for these errors and any consequent inconvenience to authors and readers.

